# Obtaining SF-6D utilities from FACT-H&N in thyroid carcinoma patients: development and results from a mapping study

**DOI:** 10.3389/fendo.2023.1160882

**Published:** 2023-08-17

**Authors:** Qing Yang, Deyu Huang, Longlin Jiang, Yuan Tang, Dingfen Zeng

**Affiliations:** ^1^ Nursing Department, Sichuan Clinical Research Center for Cancer, Sichuan Cancer Hospital & Institute, Sichuan Cancer Center, Affiliated Cancer Hospital of University of Electronic Science and Technology of China, Chengdu, China; ^2^ School of Nursing, Chengdu Medical College, Chengdu, China

**Keywords:** SF-6D, FACT-H&N, thyroid carcinoma, mapping study, health utility

## Abstract

**Objective:**

There is limited evidence for mapping clinical tools to preference-based generic tools in the Chinese thyroid cancer patient population. The current study aims to map the FACT-H&N (Functional Assessment of Cancer Therapy-Head and Neck Cancer) to the SF-6D (Short Form Six-Dimension), which will inform future cost-utility analyses related to thyroid cancer treatment.

**Methods:**

A total of 1050 participants who completed the FACT-H&N and SF-6D questionnaires were included in the analysis. Four methods of direct and indirect mapping were estimated: OLS regression, Tobit regression, ordered probit regression, and beta mixture regression. We evaluated the predictive performance in terms of root mean square error (RMSE), mean absolute error (MAE), concordance correlation coefficient (CCC), Akaike information criterion (AIC) and Bayesian information criterion (BIC) and the correlation between the observed and predicted SF-6D scores.

**Results:**

The mean value of SF-6D was 0.690 (SD = 0.128). The RMSE values for the fivefold cross-validation as well as the 30% random sample validation for multiple models in this study were 0.0833-0.0909, MAE values were 0.0676-0.0782, and CCC values were 0.6940-0.7161. SF-6D utility scores were best predicted by a regression model consisting of the total score of each dimension of the FACT-H&N, the square of the total score of each dimension, and covariates including age and gender. We proposed to use direct mapping (OLS regression) and indirect mapping (ordered probit regression) to establish a mapping model of FACT-H&N to SF-6D. The mean SF-6D and cumulative distribution functions simulated from the recommended mapping algorithm generally matched the observed ones.

**Conclusions:**

In the absence of preference-based quality of life tools, obtaining the health status utility of thyroid cancer patients from directly mapped OLS regression and indirectly mapped ordered probit regression is an effective alternative.

## 1 Introduction

Every year, many people worldwide are newly diagnosed with thyroid cancer (1), and the treatment of patients increases health care system costs. In the current context of limited health care resources, relevant health economic assessments are often required to aid in the rational allocation of health care resources. The preferred method for health economic assessment of cancer is cost-utility analysis, the standard outcome measure of which is quality-adjusted life years (QALYs), which requires the calculation of utility scores for health status (2). Preference-based generic tools are generally recommended to calculate QALYs to facilitate comparison of health outcomes across disease domains, such as EuroQoL 5-Dimensions (EQ-5D) and Short Form-6D (SF-6D) (3, 4).

Patient-reported outcome measures (PROMs) for specific diseases are used more frequently than generic tools in clinical studies. These instruments are preferentially used to study specific diseases and can reflect subtle changes in health status (5). The Functional Assessment of Chronic Illness Therapy (FACIT) and the European Organization for Research and Treatment of Cancer (EORTC) have developed groundbreaking quality of life assessment tools for cancer patients, which have been widely applied and extensively validated (6–8). The FACT-H&N is specifically used to measure quality of life in head and neck cancer patients (9). When preference-based tools are not available, “mapping” is a common approach to calculating QALYs, which can generate statistical formulas or algorithms that allow disease-specific or clinical tools to predict utility scores from generic preference-based tools and subsequently generate QALYs for cost-utility analysis in clinical studies (10). There are currently two broad mapping approaches, in which the direct approach models the health state utility value itself; the indirect approach, also called response mapping, models each dimension based on the preference scale in the first step and then calculates the predicted utility value in the second step (11).

According to the literature, most mapping studies use ordinary least squares (OLS) (10), but OLS may not be appropriate when the preference-based score is highly skewed (12). Mixed models and response mapping are also increasingly used in mapping studies (13). A systematic review identified 45 mapping studies on SF-6D, noting that the number of mapping functions for SF-6D has increased in recent years (13). Although there are studies on mapping FACT-B to SF-6D in breast cancer patients (14), and mapping FACT-G and FACT-C to SF-6D in colorectal cancer patients (15). However, apart from a mapping study from FACT-H&N to EQ-5D-5L published by our research group (16), we did not find any other mapping studies in thyroid cancer patients. To our knowledge, no study has mapped FACT-H&N scores to SF-6D utility scores using direct or indirect methods in thyroid cancer samples thus far. In this study, we used direct and indirect mapping methods to develop an optimal mapping model to map FACT-H&N from thyroid cancer patient data onto SF-6D to facilitate cost-utility analysis.

## 2 Materials and methods

### 2.1 Study design and patient population

Between May and December 2021, we conducted a cross-sectional survey at Sichuan Cancer Hospital in China. The hospital is a large tertiary grade A cancer hospital, carrying out more than 3,000 thyroid surgeries every year, and patients come from southwest China and even the whole country. The inclusion criteria for this study were as follows: (1)≥18 years of age; (2) patients with pathologically confirmed papillary thyroid carcinoma; (3) cognitive ability to understand the questionnaire; and (4) willingness to participate in this study and sign a consent form before collecting data. Patients with severe physical diseases and visual and auditory impairments were excluded from this study. The Ethics Committee of Sichuan Cancer Hospital approved the conduct of this study (reference number: SCCHEC-02-2021-061).

### 2.2 Research instruments

The instruments of this study included three questionnaires, which were the sociodemographic characteristics of the patients, SF-6D and FACT-H&N. Prior to the survey, we obtained authorization from the FACT-H&N and SF-6D development facilities. Health-related quality of life data came from two measures: the FACT-H&N and SF-6D. Demographic data and data on health-related quality of life were obtained from field surveys, whereas clinical data were obtained from electronic medical records. Data were collected by trained members of the research team, and prior to data collection, we also prepared a data collection manual to ensure the quality of data collection.

#### 2.2.1 Short form-36 health survey (SF-6D)

The SF-6D is a generic preference-based health measurement instrument that has been developed based on the SF-36 (17). There are two versions of SF-6D instruments: SF-6D version 1 (SF-6Dv1) and SF-6D version 2 (SF-6Dv2) (13, 14, 18). SF-6Dv1 comes from 11 items of SF-36v2, covering 6 dimensions (physical functioning, role limitations, social functioning, pain, mental health, and vitality), each with 4-6 levels and potentially 18,000 unique health states (19). In this study, we used the Chinese version (Hong Kong) of the SF-6Dv1 utility scoring system. This integration system has been shown to be effective with utility scores between 0.315 and 1 (20).

#### 2.2.2 FACT-H&N

FACT-H&N is a questionnaire designed by Rush University Medical Center, Chicago, USA, for functional assessment of head and neck cancer treatment. The Chinese version of the FACT-H&N has good reliability and construct validity (9) and can be used to determine the quality of life of Chinese patients with head and neck cancer. The FACT-H&N investigates the situation of patients seven days before the day of the survey, and the specific items include five domains: Physical Well-Being (PWB), Social/family Well-Being (SWB), Emotional Well-Being (EWB), Functional Well-Being (FWB), and additional concern for head and neck cancer (HNCS), with a total of 39 items. Each item is scored on a five-point scale ranging from 1 to 4 (0: not at all; 1: a little; 2: some; 3: comparable; 4: very). The five domain scores are summed to give a total scale score, which ranges from 0 to 144. Higher total scores represent better quality of life (21, 22).

### 2.3 Data analysis

This study developed the mapping functions from FACT-H&N to SF-6D using 4 modelling algorithms. These methods include OLS, Tobit, Oprobit (ordered probit regression) and beta-mixture regression models.

To date, linear regression is the most commonly used method to develop mapping models (23), which estimates parameters by minimizing the sum of squared data errors. OLS is considered the best mapping model in several studies. Given that utility metrics tend to follow a nonnormal distribution with prominent ceiling effects, OLS may have some limitations in its theoretical use to map health utilities (24). Therefore, this study explored the Tobit model, which is an alternative method to improve the ability to address ceiling effects (25). Additionally, response mapping and hybrid models are gaining popularity in developing mapping models (23, 26). In the current study, we used response mapping and mixed models in addition to linear regression and Tobit models. Response mapping is the term used for the two-stage mapping approach. Instead of modelling SF-6D utility scores directly, response mapping estimated a separate model for each of the six dimensions and calculated the probability of each of the SF-6D dimensions being at each of the four to six levels. According to these probabilities and the utility integration system of SF-6D in China, the expected SF-6D value is calculated by a mathematical method (27). In this study, oprobit regression model was used for response mapping.

Health state utility values are skewed and multimodal, there are usually a large number of observed values of 1, and there is a gap between full health and the next feasible value (28). Beta mixture regression can provide flexibility when modelling slanted, bounded preference-based measures (PBMs). This model is a two-part model composed of a polynomial logit model and beta mixture model, which is an extension of the truncated expansion beta regression model introduced by Pereira et al. (29). Currently, beta hybrid models are increasingly used in mapping environments due to their flexibility and ability to capture multiple modes (26, 28). In this study, beta mixture regression is adopted to include the full health upper limit, which is at the mass point of full health. The gap (truncation) between the upper limit and the previous feasible value (0.965) is taken into account, both with and without truncation. Although beta mixture regression models can include probabilistic masses at the lower limit of utility, this study does not include this here because our sample does not contain any observations of the lower limit of utility of PBMS. In addition, the utility value of only one sample in this study was 0.965, so the probability mass was not included at the cut-off point.

We used the same independent variables in these models to ensure that the models were comparable, each with five modelling approaches, and a two-tailed P value of less than 0.05 was considered statistically significant.

Model 1: FACT-H&N total score

Model 2: FACT-H&N total score + square term of the FACT-H&N total score

Model 3: Various domain scores for the FACT-H&N

Model 4: FACT-H&N domain scores + FACT-H&N domain scores squared

Model 5: Model 4 + age + gender

In this study, the skewness/kurtosis test was used to assess the skewness and kurtosis of SF-6D and FACT-H&N scores. The Spearman correlation coefficient was used to evaluate the correlation between FACT-H&N and SF-6D. The Spearman’s rank correlation coefficient, defined before the analysis and used to interpret the results, ranks the strength of the correlation into five levels—very weak (0–0.19); weak (0.20–0.39); moderate (0.40–0.59); strong (0.60–0. 0.79); very strong (0.80–1.00) (30).

To compare the models, the root mean square error (RMSE), mean absolute error (MAE) and mean error (AE) were used to measure the deviation between the predicted value and actual utility value. The goodness of fit was evaluated by the concordance correlation coefficient (CCC), Akaike information criterion (AIC) and Bayesian information criterion (BIC), in which higher CCC and lower AIC and BIC values indicated a better fit model. Of course, because the AIC and BIC cannot be compared with each other between direct mapping models and indirect mapping models (31), this study is only used for comparison between several models of the same kind. In the initial selection and final screening process of the optimal model, this study conducted an average ranking value (ARV), which means sorting each indicator of the model separately and calculating the average rank of these indicators (25).

First, model selection was performed according to RMSE, MAE, AE, CCC, AIC, and BIC among the four models, and the best two models in each model were selected for validation. Due to the lack of available external data in this study, two internal validation procedures were performed (32). (1) a fivefold cross-validation was used. The original sample was randomly divided into five equally sized subsamples, and of the five subsamples, one subsample was retained as validation data for testing the model, and the remaining four subsamples were used as training data. The cross-validation process was then repeated five times, and each subsample was used only once as validation data. Finally, the five results were combined to produce average ranking values (ARV). (2) 70% of the samples were randomly selected as the training set, and the remaining 30% of the samples were used as the test set to test the stability and reliability of the model. Combined with the results of each indicator in the two validation sets, the indicators are comprehensively ranked according to the ARV to select the best model. The regression model with the lowest average ranking values (ARV) was considered to be the best prediction model (10). To examine the predictive performance of the SF-6D continuum, this study estimated the best of the various models using cumulative distribution function plot. Bland-Altman plots were also used in this study to determine the width between the 95% empirical limits of agreement and to compare them to the 95% theoretical limits of agreement. Observed and predicted SF-6D values were plotted to measure the performance of the models. Following internationally accepted general guidelines proposed for instrumental mapping (33, 34).

Stata version 15.0 (StatCorp, College Station, TX) was used for data analysis except for CCC, where R4.1.1 was used. Beta mixture regression was performed using the publicly available Stata command “betamix” (35), and ordered probit regression was performed using the command “oprobit.”

## 3 Results

### 3.1 Descriptive statistics

Data were collected on a total of 1050 thyroid cancer patients. **Table 1** shows that the mean (standard deviation) age of the patient sample was 40.756 (11.330) years, with 76% females. Most patients had stage I(93.714%) or stage II(5.429%) disease. The ceiling effects existed in the health utility of 0.286% of participants. The FACT-H&N score ranged between 48 and 145 with a mean of 109.152 (SD = 15.478) and was not normally distributed (Pr (skewness) = 0.0143, Pr (kurtosis) = 0.152, p = 0.0188). SF-6D utility values ranged between 0.329 and 1, with a mean of 0.690 (SD = 0.128) and a markedly right-skewed distribution (Pr (skewness) = 0.0032, Pr (kurtosis) = 0.000, p = 0.000).

**Table 1:** Characteristics of the study sample.

Variables	Mean (SD)	Min	Max
Utility measures			
SF-6D	0.690 (0.128)	0.329	1
Flooring, *n* (%)	0(0.000)		
Ceiling, *n* (%)	3(0.286)		
FACT-N&H			
Total scores	109.152 (15.478)	48	145
PWB	22.049 (3.611)	6	29
SWB	21.346 (3.465)	8	28
EWB	19.814 (2.757)	7	24
FWB	17.587 (4.527)	2	28
HNCS	28.357 (6.635)	4	40
Socio-demographics			
Age	40.756 (11.330)	19	78
Female, *n* (%)	798 (76.000)		
TNM stage, *n* (%)			
I	984(93.714)		
II	57(5.429)		
III	5(0.476)		
IV	4(0.381)		

### 3.2 Overlap of concepts

**Table 2** shows the Spearman correlation coefficients between each dimension of the two scales (SF-6D and FACT-H&N) and the total score. The correlation coefficient between the total scores of the two scales was 0.650, indicated a strong correlation between the total scores of these two scales. The correlation coefficients between the total SF-6D scores and each dimension of the FACT-H&N ranged from 0.210 to 0.675. The correlation coefficients of the FACT-H&N total scores and the individual SF-6D items ranged from -0.247 to -0.557. The correlation coefficients of each dimension of the FACT-H&N and each entry of the SF-6D ranged from -0.063 to -0.635, except for the correlation coefficients of the total SWB score with the two entries of physical functioning and pain, for which the p values of each correlation coefficient were less than 0.05.

**Table 2:** Correlation between SF-6D and FACT-H&N scores.

Dimension	PWB	SWB	EWB	FWB	HNCS	FACT-H&N total score
Physical functioning	-0.392^*^	0.024	-0.279^*^	-0.338^*^	-0.710^*^	-0.517^*^
Role limitations	-0.469^*^	-0.242^*^	-0.467^*^	-0.395^*^	-0.276^*^	-0.467^*^
Social functioning	-0.323^*^	-0.154^*^	-0.263^*^	-0.218^*^	-0.063^*^	-0.247^*^
Pain	-0.635^*^	-0.029	-0.403^*^	-0.376^*^	-0.519^*^	-0.557^*^
Mental health	-0.438^*^	-0.123^*^	-0.462^*^	-0.269^*^	-0.102^*^	-0.336^*^
Vitality	-0.414^*^	-0.076^*^	-0.326^*^	-0.447^*^	-0.374^*^	-0.474^*^
SF-6D	0.675^*^	0.210^*^	0.572^*^	0.527^*^	0.474^*^	0.659^*^

^*^P<0.05.

### 3.3 Model development and performance

Regarding the five prediction models developed for OLS (**Table 3**), the best goodness of fit was found in Model 4 and Model 5, with RMSE, MAE, AE > 0.05 (%), AE > 0.1 (%), AIC and BIC all being low, with Model 5 having the highest CCC of 0.7189. Therefore, Model 4 and Model 5 were selected as the preferred OLS models. The bold values provided in **Table 3** were the best performing of each metric. The coefficients of the five OLS models are presented in **Table 4**.

**Table 3:** Models performance of four regression methods for mapping FACT-H&N to SF-6D utility score.

No.	Mapping method	RMSE	MAE	CCC	AE>0.05(%)	AE>0.1(%)	AIC	BIC	ARV
1	OLS M1	0.0948	0.0793	0.6732	67.52	32.10	-1964.23	-1954.31	4.71
2	OLS M2	0.0933	0.0774	0.6392	65.05	30.38	-1994.76	-1979.89	4.00
3	OLS M3	0.0890	0.0732	0.6827	62.86	27.43	-2089.38	-2059.64	2.86
4	OLS M4	0.0859	0.0691	0.6236	59.19	25.05	-2153.80	-2099.28	2.43
5	OLS M5	0.0849	0.0681	0.7189	**56.19**	**24.76**	**-2173.39**	**-2108.95**	1.00
6	TOBIT M1	0.0948	0.0793	0.6242	67.33	31.90	-1943.69	-1928.82	4.86
7	TOBIT M2	0.0933	0.0774	0.6399	64.76	30.48	-1974.83	-1955.00	3.86
8	TOBIT M3	0.0886	0.7320	0.6831	62.76	27.43	-2068.60	-2033.91	3.29
9	TOBIT M4	0.0859	0.0690	0.7112	58.19	25.24	-2133.90	-2074.42	2.00
10	TOBIT M5	0.0849	**0.0681**	**0.7195**	**56.19**	**24.67**	**-2153.72**	**-2084.33**	1.00
11	OPROBIT M1	0.0943	0.0789	0.6256	67.05	31.43	14058.30	14211.95	5.00
12	OPROBIT M2	0.0936	0.0776	0.6366	65.52	30.29	13968.27	14151.67	4.00
13	OPROBIT M3	0.0879	0.0719	0.6868	61.52	27.71	13077.45	13350.07	3.00
14	OPROBIT M4	0.0862	0.0694	0.7067	57.71	25.62	12868.87	13290.18	1.86
15	OPROBIT M5	0.0851	0.0683	0.7160	**56.19**	**24.67**	**12828.38**	13309.17	1.14
without truncation									
16	BETAMIX M1a	0.0947	0.0791	0.6278	66.95	31.90	-1195.51	-1170.72	11.14
17	BETAMIX M1b	0.0951	0.0801	0.6075	68.10	32.67	-1296.49	-1251.86	12.00
18	BETAMIX M1c	0.0950	0.0802	0.6050	68.10	32.95	-1339.02	-1274.59	11.57
19	BETAMIX M2a	0.0935	0.0775	0.6376	65.05	30.67	-1230.40	-1195.71	8.64
20	BETAMIX M2b	0.0935	0.0778	0.6382	65.14	30.76	-1232.70	-1173.23	8.93
21	BETAMIX M3a	0.0885	0.0727	0.6889	62.48	27.33	-1306.04	-1241.61	4.29
22	BETAMIX M3b	0.0891	0.0743	0.6691	63.43	28.29	-1423.25	**-1319.17**	4.57
23	BETAMIX M3c	0.0891	0.0743	0.6660	63.24	28.38	**-1461.84**	-1318.10	4.71
24	BETAMIX M4a	0.0858	0.0690	0.7100	57.71	25.14	-1386.92	-1272.92	2.57
25	BETAMIX M5a	**0.0848**	**0.0681**	0.7188	56.38	24.95	-1402.34	-1268.51	1.86
with truncation									
26	BETAMIX M1a#	0.0954	0.0793	0.6321	65.90	32.48	-988.20	-963.41	13.21
27	BETAMIX M1b#	0.0950	0.0796	0.6211	67.52	32.67	-1075.25	-1030.64	13.00
28	BETAMIX M1c#	0.0954	0.0803	0.6110	68.00	33.24	-1201.15	-1136.71	13.79
29	BETAMIX M2a#	0.0937	0.0774	0.5418	64.29	30.86	-1042.73	-1008.28	11.71
30	BETAMIX M3a#	0.0897	0.0735	0.6899	60.90	28.57	-1087.57	-1023.13	7.29
31	BETAMIX M3b#	0.0892	0.0739	0.6762	63.05	28.10	-1228.89	-1124.81	6.71

^#^with truncation.

BETAMIX Ma: 1 component without truncation; probability mass at full health.

BETAMIX Mb: 2 components without truncation; probability mass at full health.

BETAMIX Mc: 3 components without truncation; probability mass at full health.

M1 = Regression model including FACT-H&N total score as explanatory variable.

M2 = Regression model including FACT-H&N total score, square term of the FACT-H&N total score as explanatory variables.

M3 = Regression model including various domain scores for the FACT-H&N as explanatory variables.

M4 = Regression model including FACT-H&N domain scores, FACT-H&N domain scores squared as explanatory variables.

M5 = Regression model including FACT-H&N domain scores, FACT-H&N domain scores squared, age, gender as explanatory variables.The bold values provided in Table 3 were the best performing of each metric

**Table 4:** Coefficient estimates of ordinary least-square regression.

Variable	OLS M1	OLS M2	OLS M3	OLS M4	OLS M5
Constant	0.08070^***^	0.61238^***^	0.02367	0.69631^***^	0.70158^***^
FACT-H&N total score	0.00558^***^	-0.00451*			
FACT-H&N squared		0.00005^***^			
PWB			0.01563^***^	-0.00528	-0.00493
SWB			0.00226^**^	-0.00457	-0.00267
EWB			0.00870^***^	-0.01836*	-0.01987^*^
FWB			0.00422^***^	-0.00578	-0.00507
HNCS			0.00093	-0.00411	-0.00499
Dimension squared					
PWB squared				0.00051^***^	0.00049^***^
SWB squared				0.00016	0.00010
EWB squared				0.00075**	0.00079^***^
FWB squared				0.00028*	0.00025^*^
HNCS squared				0.00009	0.00010
Age					-0.00014
Gender					0.03033^***^

^*^ P<0.05.

^**^ P<0.01.

^***^ P<0.001.

In the Tobit regression (**Table 3**), the best prediction accuracy and goodness of fit were also found in Model 4 and Model 5. Compared with OLS M5, the CCC value of Tobit M5 was slightly higher, but the AIC and BIC values were also slightly higher, and the other indicators were similar to those of OLS. The coefficients of the five Tobit models are shown in **Table 5**.

**Table 5:** Coefficient estimates of Tobit model.

Variable	TOBIT M1	TOBIT M2	TOBIT M3	TOBIT M4	TOBIT M5
Constant	0.07941^***^	0.61753^***^	0.02268	0.69769^***^	0.70337^***^
FACT-H&N total score	0.00559^***^	-0.00462^**^			
FACT-H&N squared		0.00005^***^			
PWB			0.01567^***^	-0.00567	-0.00534
SWB			0.00226^**^	-0.00436	-0.00241
EWB			0.00869^***^	-0.01824^*^	-0.01978^*^
FWB			0.00425^***^	-0.00592	-0.00521
HNCS			0.00093	-0.00410	-0.00498
Dimension squared					
PWB squared				0.00052^***^	0.00050 ^***^
SWB squared				0.00015	0.00009
EWB squared				0.00074^**^	0.00078^***^
FWB squared				0.00028^**^	0.00026^*^
HNCS squared				0.00009	0.00010
Age					-0.00014
Gender					0.03053^***^

^*^P<0.05, ^**^P<0.01, ^***^P<0.001.

In the ordered probit regression, RMSE, MAE, AE > 0.05 (%), AE > 0.1 (%) and AIC of Model 5 were the lowest and CCC was the highest, except that the BIC value was slightly higher than that of Model 4. Therefore, Models 4 and 5 were selected as the preferred ordered probit regression models (**Table 3**). The coefficients of order probit regression Model 5 are listed in **Table 6**.

**Table 6:** Indirect mapping equations for each dimension from FACT-H&N to SF-6D (Ordered Probit regression): Model 5.

Variable	SF-6D dimensions					
	Physical functioning	Role limitation	Social functioning	Pain	Mental health	Vitality
PWB	0.29190^**^	0.11622	-0.09457	-.02452	-0.12182	0.07418
SWB	0.17696	0.08036	-0.23094^**^	-0.18215^*^	-0.09977	-0.13206
EWB	-0.04227	0.22319	0.21207	-0.00724	0.30961^**^	-0.07582
FWB	0.08981	0.13736^*^	0.02718	-0.00185	0.03522	-0.10139
HNCS	0.12371^*^	0.10506^*^	-0.05160	0.14267^***^	-0.13720^***^	-0.06240
Dimension squared						
PWB squared	-0.00700^**^	-0.00548^*^	-0.00101	-0.00480^*^	-0.00088	-0.00348
SWB squared	-0.00329	-0.00273	0.00513^*^	0.00531^*^	0.00220	0.00364
EWB squared	0.00129	-0.00937^*^	-0.00670^*^	-0.00014	-0.01291^***^	0.00142
FWB squared	-0.00258	-0.00523^***^	-0.00139	0.00061	-0.00123	0.00069
HNCS squared	-0.00534^***^	-0.00150	0.00168^*^	-0.00347^***^	0.00366^***^	0.00074
Age	-0.00112	0.00106	0.00113	0.00350	0.00723^*^	0.00649
Gender	0.22318^*^	-0.48082^***^	-0.09718	-0.10139	-0.25990^**^	-0.23725
/cut1	1.85943	2.15046	-3.48319	-4.36860	-5.26322	-4.44934
/cut2	2.57403	3.08038	-2.92791	-3.30444	-3.28649	-3.45617
/cut3	3.43584	3.24381	-2.02937	-2.27907	-1.52030	-2.54822
/cut4	3.92963		-1.39768	-1.45893	-0.30247	-1.13088^*^
/cut5	7.42718			-.523392		

^*^ P<0.05

^**^ P<0.01

^***^ P<0.001

Among the beta-mixture regression models, except that AIC and BIC were slightly higher in Model 5 than in Model 3, the other indicators: RMSE, MAE, AE were the lowest, and CCC was the highest. Except for Model 5, RMSE, MAE, AE in Model 4 were lower than those in the other models, while CCC was higher. Therefore, Models 4 and 5 were chosen as the preferred beta-mixture regression models (**Table 3**). The coefficients of Beta-mixture regression (Model 5) are shown in **Table 7**.

**Table 7:** Coefficient estimates of Beta-mixture model: Model 5.

sf6d	Coef.	Std. Err.	z	P>z	[95% Conf.	Interval]
C1_mu						
PWB	-0.04356	0.0417546	-1.04	0.297	-0.1254002	0.0382748
SWB	-0.03207	0.0415184	-0.77	0.44	-0.113441	0.0493081
EWB	-0.13633	0.0567716	-2.4	0.016	-0.247602	-0.0250613
FWB	-0.04477	0.0240559	-1.86	0.063	-0.0919152	0.0023821
HNCS	-0.03050	0.0204553	-1.49	0.136	-0.0705867	0.0095967
PWB2s	0.00338	0.0010156	3.32	0.001	0.0013862	0.0053671
SWB2s	0.00100	0.0010289	0.97	0.332	-0.0010179	0.0030153
EWB2s	0.00515	0.0015302	3.36	0.001	0.0021463	0.0081446
FWB2s	0.00197	0.0006968	2.83	0.005	0.0006066	0.0033379
HNCS2s	0.00068	0.000368	1.84	0.065	-0.0000435	0.001399
age	-0.00095	0.0015095	-0.63	0.527	-0.003913	0.0020042
Gender	0.19153	0.0398673	4.8	0	0.1133908	0.2696675
_cons	0.77070	0.6534747	1.18	0.238	-0.5100871	2.051487
C1_lnphi						
_cons	2.63908	0.0423821	62.27	0	2.556016	2.722151
PM_ub						
PWB	-131.36070	20926.23	-0.01	0.995	-41146.02	40883.29
SWB	375.22690	190000.6	0	0.998	-372019.1	372769.6
EWB	3973.63700	267748.8	0.01	0.988	-520804.4	528751.7
FWB	-36.15482	4941.716	-0.01	0.994	-9721.741	9649.431
HNCS	35.57312	18643.09	0	0.998	-36504.21	36575.35
PWB2s	2.91539	391.8796	0.01	0.994	-765.1545	770.9853
SWB2s	-8.38414	4136.818	0	0.998	-8116.398	8099.629
EWB2s	-87.21527	5916.043	-0.01	0.988	-11682.45	11508.02
FWB2s	1.06046	115.0786	0.01	0.993	-224.4895	226.6104
HNCS2s	-0.62937	246.1045	0	0.998	-482.9853	481.7266
age	-0.98171	102.9099	-0.01	0.992	-202.6813	200.7179
Gender	-1.52704	4512.225	0	1	-8845.326	8842.272
_cons	-48205.21000	3979138	-0.01	0.99	-7847173	7750763
C1_phi	14.00036	0.5933646			12.88438	15.213

**Figure 1:**
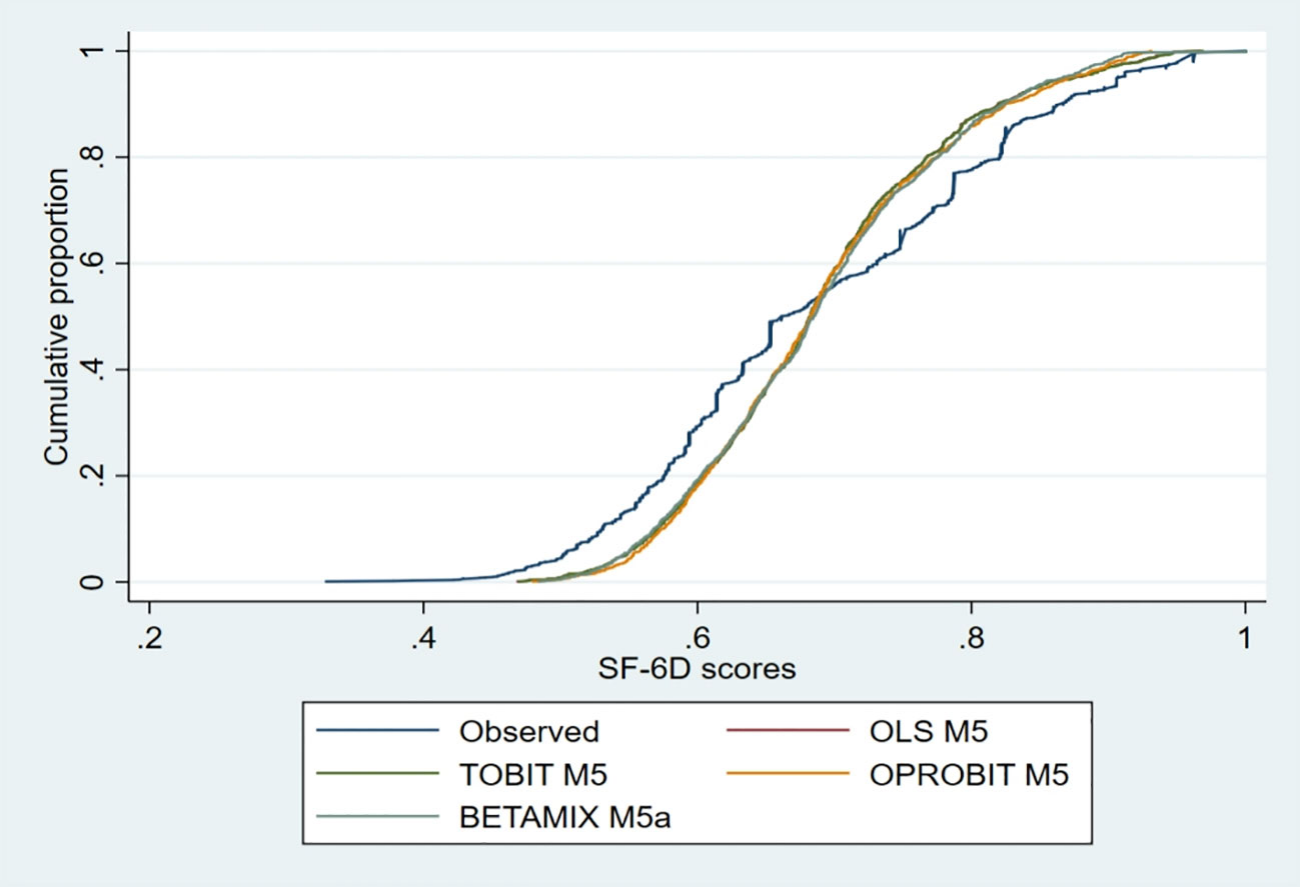
The conditional distribution function plot of the preferred models.

The OLS model predicted the mean best. Although the predicted value of OLS was closer to the observed value and OLS M4 predicted a closer minimum value, the OLS model was less effective than the beta-mixture regression model in predicting the condition with a utility value of one. For the median and P90, the ordered probit regression then showed a closer predictive value. The conditional distribution function plot shows that the observed data of SF-6D from the simulated data of the best fitting model all fit well, with some differences between the ordered probit model distribution and the upper end data of SF-6D (**Figure 1**). The predictive values of Models 4 and 5 are shown in **Table 8**.

**Table 8:** Descriptive summary of EQ-5D-5L utility index derived from observed and predicted values of best fitting models.

Model	Mean	SD	Minimum	P10	Median	P90	Maximum
Observed data	0.6893	0.1282	0.3290	0.5310	0.6615	0.8670	1.0000
OLS M4	0.6893	0.0952	0.4680	0.5732	0.6817	0.8197	0.9557
OLS M5	0.6893	0.0961	0.4688	0.5706	0.6822	0.8195	0.9670
TOBIT M4	0.6895	0.0955	0.4689	0.5729	0.6817	0.8203	0.9569
TOBIT M5	0.6895	0.0963	0.4699	0.5706	0.6823	0.8201	0.9687
OPROBIT M4	0.6912	0.0944	0.4833	0.5768	0.6803	0.8272	0.9304
OPROBIT M5	0.6911	0.0952	0.4798	0.5747	0.6827	0.8248	0.9311
BETAMIX M4a	0.6905	0.0948	0.4835	0.5705	0.6838	0.8233	1.0000
BETAMIX M5a	0.6905	0.0957	0.4841	0.5682	0.6846	0.8233	1.0000

**Figure 2:**
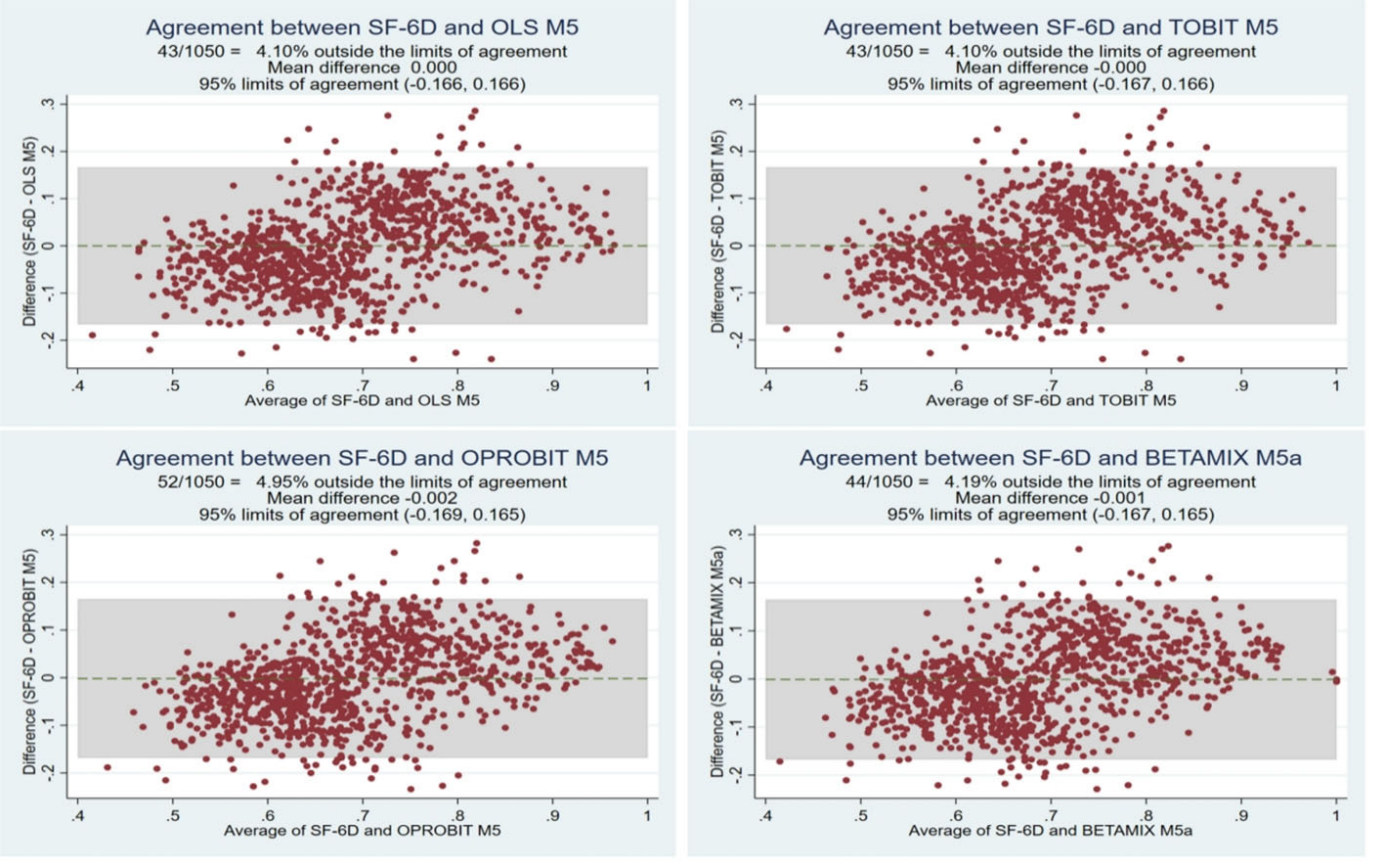
Bland-Altman plot of the observed and predicted SF-6D scores.

Bland-Altman plots showed good agreement between the observed and predicted values for SF-6D (**Figure 2**). With the exception of OLS, the actual observed values of SF-6D in Model 5 for the Tobit, ordered probit regression, and beta-mixture regression models were lower than the mean prediction scores. OLS M5 had the lowest proportion of predicted scores exceeding the 95% limits of agreement at 4.1%. A cross-validation approach also revealed similar results. The predictive ability of the eight candidate best model models was tested by fivefold cross-validation as well as by randomly drawing 30% of the samples, and the measures of goodness of fit for both internal validations are shown in **Table 9**. The ARV was calculated by combining the results of the two validations, and as a result, OLS Model 5 was the best model for comprehensive ranking, followed by ordered probit regression. The CCC between the observed utility and predicted utility of SF-6D obtained in the validation sample was 0.6940 to 0.7161, indicating good agreement.

**Table 9:** Results of best fit model validation analysis.

	Validation I: 5-fold cross-validation					Validation II: Random sample (*N*315)					ARV
	(1)RMSE	(2)MAE	(3)CCC	(4) AE>	(5) AE>	(6) RMSE	(7) MAE	(8)CCC	(9)AE>	(10) AE>	
				0.05 (%)	0.1 (%)				0.05 (%)	0.1 (%)	
OLS M4	0.0869	0.0699	0.7055	58.76	25.81	0.0845	0.0686	0.7057	57.46	27.30	4.8
OLS M5	0.0866	0.0695	0.7043	56.57	25.71	0.0833	0.0676	0.7156	57.46	27.30	2.85
TOBIT M4	0.0869	0.0697	0.7061	58.19	25.14	0.0845	0.0686	0.7060	57.46	27.30	4.15
TOBIT M5	0.0872	0.0695	0.7049	56.48	25.90	0.0833	0.0676	0.7159	57.46	27.30	3.05
OPROBIT M4	0.0874	0.0703	0.6940	58.19	26.29	0.0849	0.0690	0.7075	57.46	27.62	6.3
OPROBIT M5	0.0864	0.0695	0.7029	57.14	26.29	0.0839	0.0682	0.7161	54.92	26.98	2.95
BETAMIX M4a	0.0878	0.0693	0.6965	57.62	26.00	0.0909	0.0728	0.7024	58.10	30.79	6.6
BETAMIX M5a	0.0882	0.0684	0.6972	56.95	26.38	0.0877	0.0699	0.7126	57.14	28.25	5.3

### 3.4 Regression coefficient

The regression coefficients of the OLS and Tobit models are shown in **Tables 4**, **5**, and the regression coefficients of ordered probit Model 5 and beta-mixture regression Model 5 are shown in **Tables 6**, **7**. In OLS, Tobit model and beta-mixture regression, the square coefficients of PWB, EWB and FWB of FACT-H&N were all positive and statistically significant (p < 0.05). FACT-H&N scores in all fields were predictors of parts of the ordered probit model. Two sociodemographic variables (age and gender) were considered. Age was only significant in the mental health domain of the ordered probit model (p< 0.05), while gender was statistically significant in all four mapping models (p< 0.001).

## 4 Discussion

To our knowledge, this is the first study to map FACT-H&N scores to the common utility score SF-6D in Chinese patients with differentiated thyroid cancer. In the current study, four different regression methods and five model specifications were explored to develop the mapping function of FACT-H&N to SF-6D, which includes direct and indirect mapping. These findings provide evidence that different predictive models should be used to map SF-6D in Chinese differentiated thyroid cancer samples. In this study, SF-6D was used to measure the health utility value of thyroid cancer. It was found that only 0.286% of patients had ceiling effect, which was lower than the results obtained by our research group using the EQ-5D-5L scale (9.62%) (16), and also lower than the ceiling effect of the EQ-5D-5L scale used in the breast cancer study (3.85%) (36). This may be because SF-6D has more states than EQ-5D. Previous studies have also shown that in subgroups with better health conditions, EQ-5D often produces higher utility (ceiling effect) than SF-6D (37, 38), which is consistent with our research results.

The results of the mapping model analysis established in this study showed that the SF-6D utility score of patients with differentiated thyroid cancer in our sample was best predicted by the OLS model, followed by the ordered probit regression model. This included the total score of each dimension of the FACT-H&N, the square term of the total score of each dimension, and covariates including age and gender (Model 5). The mapping algorithm of this study combined clinical measurement tools for differentiated thyroid cancer as well as key demographic characteristics including age and gender (39, 40). Although previous mapping studies have also added covariates such as affected joints (41) and Charlson comorbidity index (42), considering that other disease related variables may not be included in the study when the algorithm of this study is used in the future, this study mainly considers the age and gender in demographics variables. In addition, after adding age and gender to the covariates, the optimal mapping model in this study achieved good predictive performance and can be used for economic evaluation in clinical research and drug clinical trials.

Currently, thyroid cancer has only been studied for health utility values, and there are no relevant mapping studies (43). Past research has shown that mapping is more likely to succeed if two tools overlap conceptually (44). In this study, the correlation coefficient between the total scores of the two scales was investigated as 0.650 by Spearman’s rank correlation before mapping. Except for the correlation coefficient between SWB total score and physical functioning and pain, the P values of the total score of the other two scales as well as the domain correlation coefficient score were less than 0.05. This may be due to the lack of domains related to social functioning of the SF-6D. SWB was also statistically insignificant in a previous mapping study that included the SF-6D for lung cancer, colorectal cancer, and breast cancer (12). In this study, we also found that SWB was not a predictor in multiple models during subsequent mapping model development. The introduction of a squared term was found to be beneficial in improving the performance of the model in this study, which suggests that the association between the two measurement tools is nonlinear (45, 46).

In the current study, model selection was primarily determined by measures of goodness of fit including RMSE, MAE, AE, CCC, AIC and BIC. In order to comprehensively consider various indicators of goodness of fit, this study also used ARV to comprehensively rank these indicators for model selection (25). Usually, models with lower ARV also mean that the various indicators of the model are better. **Table 3** shows that RMSE (0.0849), MAE (0.0681), AE > 0.05 (56.19%), AE > 0.1 (24.76%) and CCC (0.7189) of OLS M5 in the full sample of the final model obtained similar index values during the internal validation. In general, our MAE values for SF-6D were lower than those commonly reported in the literature (up to 0.19) (10).

A recent systematic review showed that the OLS model was most commonly used in 147 studies mapping EQ-5D, exceeding 75% (13). Because the OLS estimator minimizes the sum of squared errors, OLS may show the lowest RMSE, and OLS will be selected as the best model when RMSE is used as a criterion (23). The study adopted CCC to correlate the mapped utility value with the observed value, and the CCC between the observed utility and predicted utility of SF-6D obtained in the validation sample was 0.6940 to 0.7161, indicating good agreement. This coefficient is slightly higher than that in previous studies (40, 47).

The selection of the best model should not focus on only one fit index but should consider descriptive statistics of the overall goodness-of-fit index and the predicted score. Therefore, the model also considers the predictive power of the model in predicting the mean score at the time of selection. In the current study, the mean predicted SF-6D values based on OLS regression were consistent with their mean observed values. Among the four regression methods, the mean values of the predicted values based on the Tobit model, beta-mixture regression model and ordered probit regression model all produced larger predicted values than observed values. The Bland Altman plot indicated that the optimal models had similar patterns for the differences between observed and predicted values, which had been observed in published cartographic literature (48). That is to say, these models underestimated utilities at higher values and overestimated utilities at lower values. Meanwhile, Bland-Altman plots showed good agreement between observed and predicted values for SF-6D, with OLS M5 having the lowest proportion of predicted scores exceeding the 95% limit of agreement of 4.1%, a result that is similar to previous mapping models (49).

There are some limitations to the current study. First, the utility value of the SF-6D is based on the utility integration system in Hong Kong, China, because a value set suitable for mainland Chinese populations has not been developed at the beginning of this study. Therefore, the results might have been different if we had used a new value set. Second, this study suggests further validation of current mapping results using external datasets.

In conclusion, we provided algorithms to convert FACT-H&N scores into utility scores, which are readily applicable in the clinical setting when SF-6D data are unavailable. The current study provides clinicians and researchers with important evidence about the mapping algorithm that can be used in health economic evaluations of treatments and interventions for patients with differentiated thyroid cancer in China.

## Data availability statement

The raw data supporting the conclusions of this article will be made available by the authors, without undue reservation.

## Ethics statement

The study involving human participants was reviewed and approved by the Ethics Committee of Sichuan Cancer Hospital (approval number: SCCHEC-02-2021-061). All patients signed informed consent forms.

## Author contributions

Data collection and analysis were performed by YT, DZ and LJ. The first draft of the manuscript was written by QY and DH. All authors agree to be accountable for the content of the work. All authors contributed to the article and approved the submitted version.
